# Distance functions of carabids in crop fields depend on functional traits, crop type and adjacent habitat: a synthesis

**DOI:** 10.1098/rspb.2023.2383

**Published:** 2024-01-10

**Authors:** Fabian A. Boetzl, Douglas Sponsler, Matthias Albrecht, Péter Batáry, Klaus Birkhofer, Michal Knapp, Jochen Krauss, Bea Maas, Emily A. Martin, Clélia Sirami, Louis Sutter, Colette Bertrand, Aliette Bosem Baillod, Gerard Bota, Vincent Bretagnolle, Lluís Brotons, Thomas Frank, Moritz Fusser, David Giralt, Ezequiel González, Anouschka R. Hof, Henryk Luka, Ronan Marrec, Michael A. Nash, Katherina Ng, Manuel Plantegenest, Brigitte Poulin, Gavin M. Siriwardena, Teja Tscharntke, Matthias Tschumi, Aude Vialatte, Laura Van Vooren, Muhammad Zubair-Anjum, Martin H. Entling, Ingolf Steffan-Dewenter, Jens Schirmel

**Affiliations:** ^1^ Department of Ecology, Swedish University of Agricultural Sciences, Uppsala SE-750 07, Sweden; ^2^ Department of Animal Ecology and Tropical Biology, Biocenter, University of Würzburg, Am Hubland, Würzburg 97074 Germany; ^3^ Agroecology and Environment, Agroscope, Reckenholzstrasse 191, Zurich 8046, Switzerland; ^4^ ‘Lendület’ Landscape and Conservation Ecology, Institute of Ecology and Botany, HUN-REN Centre for Ecological Research, 2163 Vácrátót, Alkotmány út 2-4, Hungary; ^5^ Department of Ecology, Brandenburg University of Technology Cottbus-Senftenberg, Cottbus 03046, Germany; ^6^ Faculty of Environmental Sciences, Czech University of Life Sciences Prague, Kamýcká 129, Praha-Suchdol 165 00, Czech Republic; ^7^ Department of Botany and Biodiversity Research, Division of Biodiversity Dynamics and Conservation, University of Vienna, Rennweg 14, 1030 Vienna, Austria; ^8^ Department of Animal Ecology, Justus Liebig University Giessen, Heinrich-Buff-Ring 26-32, 35392 Giessen, Germany; ^9^ UMR Dynafor, INRAE, Toulouse University, 31326 Castanet Tolosan, France; ^10^ LTSER Zone Atelier « PYRÉNÉES GARONNE », 31320 Auzeville-Tolosane, France; ^11^ Plant-Production Systems, Agroscope, Route des Eterpys 18, 1964 Conthey, Switzerland; ^12^ Université Paris-Saclay, INRAE, AgroParisTech, UMR EcoSys, 91120 Palaiseau, France; ^13^ INRAE, Institut Agro, ESA, UMR BAGAP, 35042 Rennes, France; ^14^ Research Institute of Organic Agriculture (FiBL), Ackerstrasse 113, Postfach, Frick 5070, Switzerland; ^15^ Landscape Dynamics and Biodiversity Program, Forest Science and Technology Centre of Catalonia (CTFC), Crtra. Sant Llorenç de Morunys, km 2, 25280 Solsona, Spain; ^16^ CEBC, UMR 7372, CNRS & La Rochelle Université, 79360 Villiers-en-Bois, France; ^17^ LTSER ‘Zone Atelier Plaine & Val de Sèvre’, CNRS, 79360 Villiers-en-Bois, France; ^18^ CREAF, Cerdanyola del Vallès 08193, Spain; ^19^ CSIC, Cerdanyola del Vallès 08193, Spain; ^20^ Institute of Zoology, University of Natural Resources and Life Sciences, Vienna 1180, Austria; ^21^ iES Landau, Institute for Environmental Sciences, Ecosystem Analysis, University of Kaiserslautern-Landau, Fortstrasse 7, Landau 76829, Germany; ^22^ Instituto Multidisciplinario de Biología Vegetal (CONICET-Universidad Nacional de Córdoba), Av. Velez Sarsfield 1611, 5000 Córdoba, Argentina; ^23^ Wildlife Ecology and Conservation Group, Wageningen University, Droevendaalsesteeg 3, 6708 PB, Wageningen, the Netherlands; ^24^ Ecologie et Dynamique des Systèmes Anthropisés (EDYSAN, UMR CNRS 7058), Université de Picardie Jules Verne, Amiens, France; ^25^ Department of Ecology, Environment & Evolution, School of Life Science, La Trobe University, Bundoora, Victoria 3086, Australia; ^26^ Fenner School of Environment and Society, The Australian National University, Canberra, Australia; ^27^ Institut Agro, Univ Rennes1, INRAE, IGEPP, 35000 Rennes, France; ^28^ Tour du Valat Research Institute for the conservation of Mediterranean wetlands, Le Sambuc, 13200 Arles, France; ^29^ British Trust for Ornithology, The Nunnery, Thetford, Norfolk IP24 2PU, UK; ^30^ Agroecology, Department of Crop Science, University of Göttingen, Göttingen, Germany; ^31^ Swiss Ornithological Institute, Seerose 1, CH-6204 Sempach, Switzerland; ^32^ Faculty of Bioscience Engineering, Department of Forest and Water Management, Forest & Nature Lab, Ghent University, Geraardsbergsesteenweg 267, 9090 Gontrode, Belgium; ^33^ Department of Zoology & Biology, Faculty of Sciences, Pir Mehr Ali Shah Arid Agriculture University Rawalpindi, Rawalpindi, Pakistan

**Keywords:** distance decay, ecosystem services, ground beetles, natural pest and weed control, species spillover, sustainable agriculture

## Abstract

Natural pest and weed regulation are essential for agricultural production, but the spatial distribution of natural enemies within crop fields and its drivers are mostly unknown. Using 28 datasets comprising 1204 study sites across eight Western and Central European countries, we performed a quantitative synthesis of carabid richness, activity densities and functional traits in relation to field edges (i.e. distance functions). We show that distance functions of carabids strongly depend on carabid functional traits, crop type and, to a lesser extent, adjacent non-crop habitats. Richness of both carnivores and granivores, and activity densities of small and granivorous species decreased towards field interiors, whereas the densities of large species increased. We found strong distance decays in maize and vegetables whereas richness and densities remained more stable in cereals, oilseed crops and legumes. We conclude that carabid assemblages in agricultural landscapes are driven by the complex interplay of crop types, adjacent non-crop habitats and further landscape parameters with great potential for targeted agroecological management. In particular, our synthesis indicates that a higher edge–interior ratio can counter the distance decay of carabid richness per field and thus likely benefits natural pest and weed regulation, hence contributing to agricultural sustainability.

## 1. Introduction

Crop pests destroy a substantial share of agricultural production causing a gap between current and potential yields [1,2]. While the majority of farmers aim to control crop pests conventionally via the application of pesticides, a significant share of crop pests and weeds is regulated by natural enemies [3,4]. In particular, the richness of natural enemies plays a pivotal role for the regulation of pests and yield stability [5]. Supporting and fostering this natural pest and weed regulation via agroecological management might be a sustainable way to close yield gaps, as it can replace artificial inputs in agroecosystems while benefitting biodiversity and contributing to the pesticide reduction goal of the farm-to-fork strategy of the European Green Deal [6].

However, in order to support and exploit the potential of natural pest and weed regulation, their drivers at different spatial scales need to be understood. Recent syntheses have focussed on landscape-scale drivers and showed that increased heterogeneity (i.e. an increase in crop diversity, semi-natural habitat cover or edge density) is often beneficial for natural enemies and natural pest regulation [7–10]. In addition to these landscape-scale factors, local drivers can influence natural pest regulation and natural enemy distributions at the field scale [11] and may interact with larger-scale processes [12].

One important local factor is the within-field distribution of natural enemies in relation to the distance to field edge. Crop field edges usually undergo less intensive management and differ from field interiors due to both abiotic and biotic spillover from adjacent habitats [13]. Compared to crop field interiors, crop field edges are characterized by a higher vegetation diversity and lower yields [14,15]. Mobile natural enemies can cross habitat borders and export the ecosystem services they provide into and change species assemblages in adjacent habitats [16]. Consequently, proximity to field edges was shown to be an important driver altering natural enemy assemblages [17,18] and enhancing pest regulation [11,19]. However, reported distance functions differ among organism groups and case studies, from steep distance decays [17,20,21] over no decrease [22,23] to increases [22,24] in natural enemy responses towards the field interior, indicating more complex relations (see electronic supplementary material, figure S1). While causes of these discrepancies remain unclear, crop type and adjacent habitats seem particularly relevant.

Different crop types vary in associated management practices (e.g. tillage intensity), as well as the vegetation biomass and cover they provide overall, and during different periods of the growing season. Vegetation biomass influences food resources available for natural enemies through changes in pest biomass. Vegetation cover determines evapotranspiration, remaining soil moisture content and soil temperature [25], and may affect natural enemy distributions and activity due to species-specific microclimate and habitat preferences.

In agricultural landscapes, adjacent non-crop habitats such as grassy field margins, flower strips or hedgerows are commonly established next to crop fields to benefit biodiversity [26]. Adjacent non-crop habitats, including forest remnants, alter microclimate and soil nutrient levels in the edges of neighbouring crop fields, potentially affecting natural enemy assemblages [27,28]. Many natural enemies also depend on the presence of such little-disturbed habitats for overwintering, shelter during crop management and alternative food resources [29,30]. Different types of adjacent habitats support distinct communities of natural enemies with varying species and trait compositions [31,32]. Their presence could thus enrich natural enemy assemblages in adjacent crop fields via spillover [16] and increase pest regulation efficiency.

Optimizing landscape composition and configuration for local natural pest regulation requires knowledge about the effects of different habitat types and configurations on natural enemies. Available case studies on within-field distance functions of natural enemies usually investigated single crop types and adjacent habitats resulting in the necessity of a synthesis. By compiling multiple case studies into a unified database, a comprehensive synthesis allows evaluation of potential modulation of distance functions by crop type identity and adjacent habitat identity. Especially for weak-dispersing ground-dwelling natural enemies, which are important natural pest regulation agents, within-field distance may also lead to a filtering of assemblages based on certain traits and dispersal abilities [33]. The possibility of functional traits modulating distance functions has rarely been considered with very few studies including functional traits in specific contexts [17,22,34].

Here, we report the results of a quantitative synthesis aimed at unveiling the drivers of within-field distance functions of natural enemies targeting carabids. We focussed on a combination of three potential drivers, crop types, adjacent habitats and functional traits of the species, to close gaps left by previous studies. We chose carabids as they are one of the most studied natural enemy taxa due to their diversity and important role in natural pest and weed regulation [35,36]. Due to their importance, a high number of datasets is available and we have good knowledge about the ecology of the species in Europe, which made it possible to analyse functional traits. For our analyses, we focussed on two functional traits directly related to natural pest and weed regulation: diet preferences (granivores and carnivores) and body size, an indicator for metabolic rates and thus consumption rates [37,38] and of dispersal capability.

**Figure 1.**
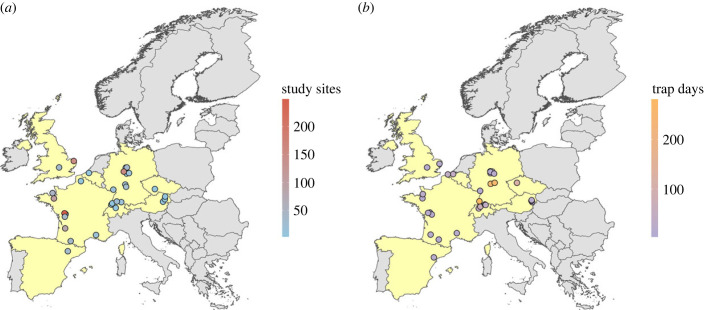
Map of European countries from which datasets were used for the analyses (yellow coloration). Coloration of the points indicates the number of study sites available in each dataset as a measure of replication *(a*) and the sampling duration covered in each dataset, i.e. the total number of days a single pitfall trap was active (trap days) as a measure of sampling effort (*b*). Points jittered by 0.5° to reduce overlay. For more information on the datasets, see electronic supplementary material, table S2.

We gathered, harmonized and re-analysed 28 published datasets (figure 1) on within-field distance functions of carabids, covering 1204 study sites with 2768 unique within-field sampling plots and data of 345 603 carabid individuals belonging to 241 species in five crop types (cereals, legumes, maize, oilseed crops and vegetables) using hierarchical Bayesian generalized linear regression models. We aimed at answering the following questions: (i) are there general within-field distance functions of carabid richness and activity densities? (ii) Does the magnitude and direction of distance functions depend on crop type and adjacent habitat type? (iii) Do the observed distance functions depend on carabid traits (diet and body size)?

## 2. Material and methods

### (a) Database creation and data handling

Prior to searching for suitable datasets, we identified data inclusion criteria needed for our analyses. Suitable datasets had to specify: (i) at least two sampling plots within a crop field at different, spatially referenced distances from a field edge, (ii) crop type and type of adjacent habitat, (iii) carabid assemblage characteristics (species richness and/or activity densities), (iv) at least three independent study sites (i.e. crop fields, minimum distance 500 m between sites) and (v) a minimum of 7 days of sampling (i.e. 7 days of active pitfall trapping). To obtain suitable datasets, we conducted a literature search in the ISI Web of Science Core Collection, screened all resulting publications for suitability and contacted authors of potentially suitable publications (331 publications prior to article screening, thereof 44 publications met our selection criteria after full-text filtering of which 18 datasets were obtained; see electronic supplementary material for search terms and electronic supplementary material, table S1 for results). For some older publications, data were unfortunately not available. We additionally asked scientists working on carabid distance effects for additional suitable, published or unpublished datasets. In total, we had access to 35 suitable datasets (28 from Europe, seven from outside Europe). Due to the limited availability of datasets from outside Europe and the considerable geographical range of these datasets across different continents and ecosystems, as well as the unavailability of data on species traits, we decided to limit our analyses to the 28 datasets from European agroecosystems that met our selection criteria (electronic supplementary material, tables S2 and S3). Within each selected dataset, we excluded all study sites for which not all of the above-mentioned criteria were met (e.g. if a study site had only one sampling plot or suffered from trap losses resulting in fewer than 7 trap days; see definition of trap days below). The 28 datasets cover eight countries from temperate Europe and 1204 study sites with 2768 unique study site × distance combinations (sampling plots). It contains data of 345 603 carabid individuals belonging to 241 species recorded in 15 different crops (figure 1; electronic supplementary material, figure S2, tables S4 and S5).

We carefully checked all datasets for typographical errors and synchronized carabid nomenclature following Lorenz [39], to avoid species duplications. Across all datasets, sampling was limited to the main growth season until crop harvest with a clear peak in May/June (electronic supplementary material, table S2), the main peak of activity of ground dwelling arthropods in temperate Europe. Within all datasets, data were pooled (i) over all sampling intervals (wherever sampling was repeated in time) as consecutive time intervals differ due to progressing phenology, and (ii) across pitfall traps per sampling plot (i.e. if several traps were used per sampling plot with the same distance to the field edge). The total sampling effort in each plot was recorded as ‘trap days' (days a single trap was active), combining both the number of traps within the sampling plot and the temporal extent of the sampling in all traps. To avoid bias due to trap losses, we eliminated all combinations of study site and sampling interval where data were missing. Activity densities in each sampling plot were then divided by the accumulated trap days to obtain a per day activity density per trap (henceforth ‘activity density').

Carabid traits with respect to diet preference (granivorous, omnivorous and carnivorous) and mean body size (a proxy for food intake rates) were obtained from the database carabids.org (extracted in spring of 2019) [40], monographies as well as from previous studies on carabid functional traits (electronic supplementary material, table S5). We divided richness and activity densities on the basis of desired ecosystem functions between predominantly granivorous species that are likely to deliver weed regulation (henceforth granivorous) and predominantly carnivorous species that are likely to deliver animal pest regulation services (henceforth carnivorous) as well as into three different size classes (small: less than 6 mm; medium: 6–10 mm; large: greater than 10 mm) that have been used previously [17,41]. For the distribution of size classes and diet preferences within our dataset, see electronic supplementary material, table S6. Results for true omnivorous species can be found in electronic supplementary material, figures S4 and S5, tables S8 and S9. We did not include wing shape in our analyses as this trait varies between individuals in many species and even the presence of fully developed wings is neither a reliable predictor of the ability nor the willingness of a species to fly, resulting in wing shape being a bad predictor for dispersal ability.

To assess the effects of different crop types, we pooled crop types according to Redlich *et al.* [42] (largely following the FAO crop classification) into five crop types: (i) cereals (except maize), (ii) maize, (iii) oilseed crops (oilseed rape and sunflower), (iv) vegetables and (v) legume crops. To assess the effects of adjacent habitat types beyond those of narrow grassy field margins that can be assumed to be present everywhere, we categorized adjacent habitats across studies into three categories: (i) herbaceous adjacent habitats (open habitats with short, grassy or herbaceous vegetation such as sown grassy margin strips, flower strips, flowering fields and semi-natural grasslands), (ii) woody adjacent habitats (hedgerows, forests) and (iii) no adjacent non-crop habitats (other crop fields, roads; henceforth control).

### (b) Statistical analyses

We modelled the response variables richness (number of species) and activity density (both separately for diet classes, the latter also for body-size classes) using hierarchical Bayesian generalized linear regression models, with a separate model specified for each response variable. Models for richness and all responses linked to traits excluded carabids with no species level identification (1969 individuals or approx. 0.57%). In describing the specification of our models, we followed the definitions of ‘constant' and ‘varying' effects proposed by Gelman [43] as an alternative to the more commonly used ‘fixed' and ‘random' effects. In all models, distance from crop field edge (continuous variable, distance), ‘crop type' (five types as defined above), ‘adjacent habitat type' (herbaceous, woody or control), and the interaction terms ‘distance' × ‘crop type' and ‘distance’ × ‘adjacent habitat type' were specified as constant effects. We used ‘distance' as a varying slope nested within site, allowing the slope of distance to vary within each site which is a conservative approach that reduces false positive results [44] and allows for a possible modulation of distance functions by other, unknown factors (among others landscape composition or management differences) across sites. We used ‘site' nested within ‘study' as a varying intercept accounting for study-specific methodological differences and site-specific differences affecting intercepts (e.g. landscape context). Quantifying potential effects of landscape context on intercepts was neither a focus of this study nor was it possible due to the low availability of detailed landscape data. To account for different sampling effort across studies in richness models, we included log-transformed ‘trap days' as a constant effect, as we expected richness to saturate with increasing sampling effort.

For richness, we used the Poisson error distribution with a log link function. For activity density, which is continuously positive and open-bounded at zero, we opted for a hurdle gamma model with a log link function (electronic supplementary material, table S7). To stabilize model fitting, distance was downscaled by a factor of 100, but parameter estimates were rescaled post-fitting to represent 10 m units for predictions. Marginalized distance slope estimates were obtained using the package ‘emmeans' version 1.7.3 [45]. We regarded slope estimates as true increases or decreases if a slope of 1 (i.e. a flat slope indicating no change with distance) was not included in at least 95% of the posterior predictions (henceforth 95% CI). We present the slope estimates (as percentage of change for every 10 m) together with the posterior probability (Pr) for a positive/negative slope (i.e. increase or decrease; the proportion of all positive/negative slope estimates among the posterior predictions). A detailed elaboration of our statistical methodology including the software used and the R code for our modelling and validation workflow can be found in electronic supplementary material, S2–S4.

Most studies included in our dataset predominantly covered a limited subset of crop types and adjacent habitat types with highly heterogeneous sampling efforts (electronic supplementary material, figure S2 and table S2). In addition, intercepts in the individual datasets are likely to be affected by different aspects of the surrounding landscapes (see e.g. [9]). Therefore, caution is advised when comparing differences in intercept values among studies. Due to this heterogeneity within the dataset, we focus on comparing slope effects (i.e. distance functions) over intercept effects.

## 3. Results

Here, we report and compare marginalized slope estimates, as a percentage of change in the response for every 10 m from the field edge, together with the posterior probability (Pr) for a positive or negative slope, i.e. increase or decrease in the response with increasing distance to the field edge (electronic supplementary material, table S9). All increases and decreases in responses can be extrapolated across the within-field distance gradients in multiples of 10 m by

3.1 $$\begin{array}{rl} & \mathrm{percentage}\;\mathrm{at}\;\mathrm{distance}\\ & \;=100\;\text{\%}\;\times {\left(\mathrm{slope}\;\mathrm{estimate}\right)}^{\mathrm{distance}\;\mathrm{in}\;\mathrm{multiples}\;\mathrm{of}\;\;10\;m}. \end{array}$$

### (a) Distance functions across all crop types and adjacent habitat types

**Figure 2.**
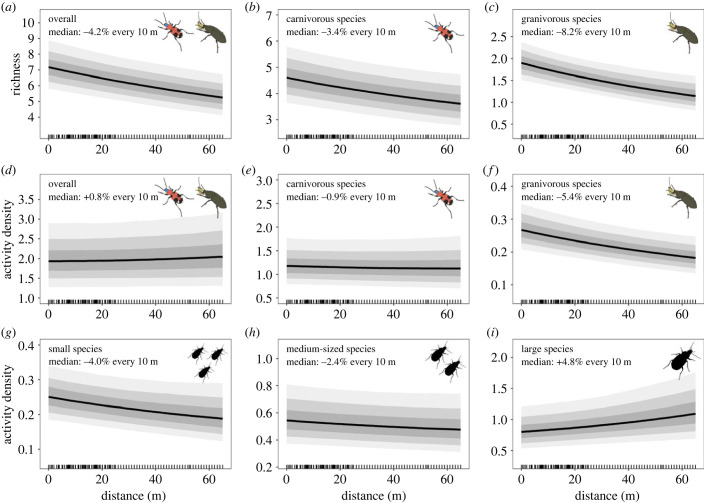
Overall marginal predictions for (*a*) overall carabid richness, (*b*) richness of carnivorous species, (*c*) richness of granivorous species, (*d*) overall activity density, (*e*) activity density of carnivorous species, (*f*) activity density of granivorous species, (*g*) activity density of small carabids (less than 6 mm body size), (*h*) activity density of medium-sized carabids (greater than 6 mm but less than 10 mm body size) and (*i*) activity density of large carabids (greater than 10 mm body size) across the within-field distance gradient. Response curves depict posterior predictions with 50% (dark), 80% (medium) and 95% (light) credible intervals (CI), back-transformed from the log-link scale to response scale. The rug indicates distances for which data points were available, predictions are limited to the distances covered by the available data (not all data used in the models is shown; distance was clipped at 65 m for visualization as a majority of the data was available up to this distance). For statistics and values, see electronic supplementary material, tables S8 and S9.

Across all crop and adjacent habitat types, overall carabid richness and the richness of carnivorous and granivorous carabids decreased by 4.2% (Pr = 0.95), 3.3% (Pr = 0.85) and 8.2% (Pr = 0.96), respectively, from the field edge towards the field interiors (all reported increases and decreases are changes for every 10 m; figure 2*a,b,c* electronic supplementary material, tables S8 and S9). By contrast, overall activity density and the activity density of carnivorous species remained rather constant with an increase of 0.8% (Pr = 0.57) and a decrease of 0.9% (Pr = 0.59), respectively, while the activity density of granivorous species decreased by 5.4% (Pr = 0.90; figure 2*d–f*; electronic supplementary material, tables S8 and S9). Activity density decays of carnivores and granivores were thus compensated by the few omnivorous species, predominantly one very abundant species, *Poecilus cupreus*, that increased in abundance towards the field centres (see results for omnivorous species in electronic supplementary material, figures S4 and S5, tables S8 and S9). The activity density of small and medium-sized carabid species decreased by 4.0% (Pr = 0.80) and 2.4% (Pr = 0.70), while the activity density of large species increased by 4.8% (Pr = 0.76, figure 2*g–i*; electronic supplementary material, tables S8 and S9). Carabid richness strongly increased by 59.7% per log pitfall trap days and saturation was not reached after 200 cumulative trap days (electronic supplementary material, figure S3 and table S8).

### (b) Distance functions in relation to crop type

We found that including crop type, and to a lesser extent adjacent habitat types, contributed significantly to explaining the variation in distance functions. For effects of crop type and adjacent habitats, we report median slope estimates with Pr > 0.95 (all slope estimates and ranges are reported in electronic supplementary material, table S9).

**Figure 3.**
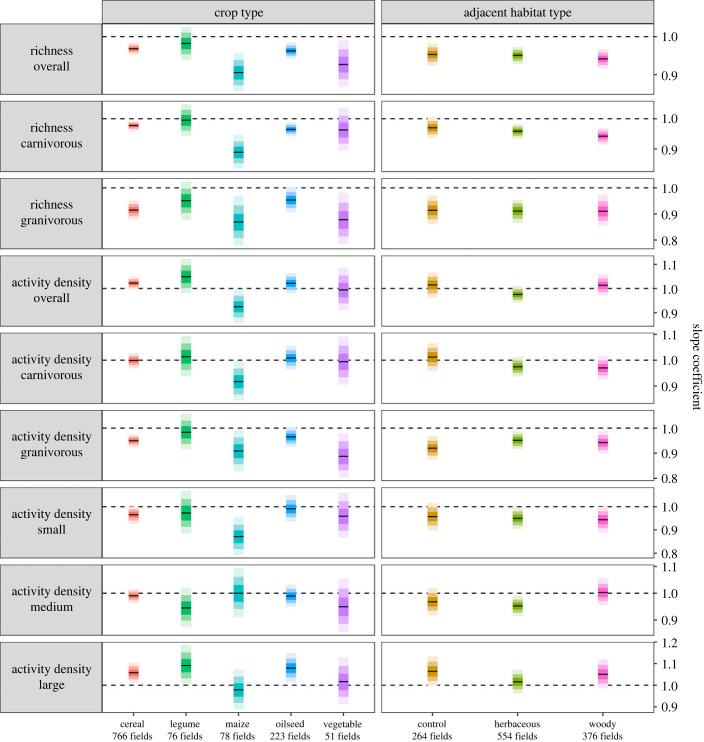
Overall marginal within-field distance slope coefficients for overall carabid richness, richness of predominantly carnivorous species, richness of predominantly granivorous species, overall activity density, activity density of predominantly carnivorous species, activity density of predominantly granivorous species, activity density of small carabids (less than 6 mm body size), activity density of medium-sized carabids (greater than 6 mm but less than 10 mm body size) and activity density of large carabids (greater than 10 mm body size; from top to bottom) in relation to crop type (left) and adjacent habitat type (right). Slope coefficient estimates (median) of the posterior sample (black lines) with 50% (dark), 80% (medium) and 95% (light) credible intervals (CI), back-transformed from the log link scale to response scale. The dashed line represents the slope coefficient of 1, i.e. a flat slope. Coefficients can be interpreted as per cent change in the response every 10 m towards the field centre. For statistics and values, see electronic supplementary material, tables S8 and S9.

Overall carabid richness decreased along the within-field distance gradient in all crop types but legumes, with the strongest decreases in maize and vegetables, at 9.5% and 7.3%, respectively (figure 3; electronic supplementary material, tables S8 and S9). Richness of granivorous species decreased in cereals, maize, oilseed crops and vegetables by 8.6%, 13.3%, 4.7% and 12.4%, respectively, while decreases in the richness of carnivorous species were smaller and occurred in cereals, maize and oilseed crops, at 2.2%, 11.2% and 3.6% respectively (figure 3; electronic supplementary material, tables S8 and S9).

Overall carabid activity density was relatively stable or increased in most crops within the field but decreased in maize by 7.5% (figure 3; electronic supplementary material, tables S8 and S9). While the activity density of carnivorous species decreased only in maize (8.4%), the activity density of granivorous species decreased along the within-field distance gradient in cereals (5.0%), maize (9.3%), oilseed crops (3.6%) and vegetables (11.3%; figure 3; electronic supplementary material, tables S8 and S9). The activity density of small species decreased within the field in cereals (3.4%) and maize (12.9%) and the activity density of large species increased in cereals (5.7%), legumes (9.0%) and oilseed crops (7.9%; figure 3; electronic supplementary material, tables S8 and S9). The activity density of medium-sized species was not affected by within-field distance in any crop type (figure 3; electronic supplementary material, tables S8 and S9).

### (c) Distance functions in relation to adjacent habitat type

Overall carabid richness decreased from all adjacent habitat types towards field interiors with the steepest decrease of 5.8% in fields bordering woody habitats (figure 3; electronic supplementary material, tables S8 and S9). The richness of carnivorous species decreased in fields bordering herbaceous (4.2%) or woody habitats (5.8%) as well as in control fields (3.0%) but richness decreases were much steeper in granivorous species, at 8.8%, 9.1% and 8.7% in fields bordering herbaceous or woody habitats and control fields not bordering semi-natural habitats, respectively (figure 3; electronic supplementary material, tables S8 and S9). Overall carabid activity density and the activity density of carnivorous species remained relatively constant in all fields bordering any adjacent habitat type and control fields (figure 3; electronic supplementary material, tables S8 and S9). By contrast, the activity density of granivorous species decreased from all adjacent habitat types towards the field interiors, with the strongest reductions in control fields (8.1%) and fields bordering woody habitats (5.8%; figure 3; electronic supplementary material, tables S8 and S9). The activity density of small species decreased in fields bordering herbaceous (5.0%) or woody habitats (5.6%), the activity density of medium-sized species decreased in fields bordering herbaceous habitats (4.8%), while the activity densities of large species increased by 6.4% along the distance gradient in control fields (figure 3; electronic supplementary material, tables S8 and S9).

## 4. Discussion

Understanding the local drivers of the within-field distance functions of natural enemies is essential for adapting natural pest and weed regulation and reducing insecticide and herbicide inputs in agroecosystems. In a quantitative synthesis of 28 datasets and 1204 study sites across eight European countries, we found that carabid richness strongly decreased from field edges towards field interiors, whereas overall carabid density slightly increased. Moreover, crop types, functional traits of carabids, and, to a lesser extent, adjacent habitat types explained variation in within-field distance functions.

### (a) Within-field distance functions of carabids

Crop type was a major driver of distance functions of carabids, with distance slopes varying considerably across crop types. This implies that crop phenology and physiognomy, as well as management regimes associated with certain crop types (i.e. soil disturbance, vegetation and microclimate), strongly affect natural enemy distributions in agroecosystems [46]. Maize and vegetables showed the steepest decreases of carabid richness, with less than 68% of the species remaining at 50 m distance from the edge. Maize was also the only crop where we observed a decrease in overall carabid activity density towards the field interiors, with particularly strong decreases of granivorous and small species, which indicates that maize is a rather hostile crop for carabids. This could be due to intensive management, including the widespread use of herbicides and deep ploughing for weed regulation, which minimizes food resources towards field interiors, especially for weed seed-eating carabids [47]. Another reason might be the phenology of these crops. For instance, maize is sown in spring, which limits the availability of resources early in the season and may hamper the establishment of carabid populations. Carabid richness also decreased towards field interiors in cereals and oilseed crops, but reductions were less pronounced, with more than 82% of the species remaining 50 m from the field edge. Legumes were the only crop type in which we did not observe a steep decrease in carabid richness towards field interiors. This indicates that, for some carabid species, legumes, and to a lesser extent cereals and oilseed crops, offer more suitable habitat conditions than maize and vegetables.

By contrast, carabid activity densities of carnivorous species remained rather constant or even increased towards field interiors in the case of large, mainly carnivorous species, while only activity densities of small and granivorous species clearly decreased across most crops. Apart from management and microclimate, prey availability is likely a major bottom-up limitation for these natural enemies. Arable weeds as seed resources for granivorous species generally decrease towards the field interiors [47], while pests consumed by carnivorous species can be assumed to increase concomitantly due to increased crop density and productivity [14]. Both factors are also likely to vary with crop type and associated management. Unfortunately, weed and (conclusive) pest assessments were not available for the majority of the datasets analysed here. More parallel assessments of the spatial distribution of beneficial arthropods and pests are necessary to clarify trophic drivers of distance functions.

The observed increase in activity densities of large species (of which 91% were carnivorous or omnivorous) in cereals, legumes and oilseed crops may result in higher overall prey consumption as metabolic rates increase with body size [37]. However, prey spectra change with predator body size, and larger predators are more likely to engage in intraguild predation [48,49]. This could indicate that, while some larger pests may be regulated more effectively towards field interiors, it may narrow the overall breadth of the pest spectrum regulated. Moreover, even with increasing activity densities, the richness of carnivorous carabids usually decreased towards the field interiors, which impoverishes assemblages and may lead to a loss of functional diversity, redundancy and resilience [50].

Adjacent herbaceous and woody habitats affected within-field distance functions less than different crop types. The presence of herbaceous habitats could generally not buffer distance decay effects in carabid richness compared to the control fields. Similarly, distance functions in fields adjacent to woody habitats were comparable to those in the control fields indicating that adjacent woody habitats are not beneficial for carabids during the growing season. Woody habitats are, however, important earlier in the season as their edges provide overwintering sites for carabids [34,51] and other natural enemies like spiders [52], and thus facilitate colonization of crop fields early in the season. We did, however, not observe lasting effects later in the growing season, which is when the datasets were collected.

The limited effects of adjacent habitats on carabid richness and density reported here may arise from interactive effects between adjacent habitats and crop types or non-crop habitat sizes or qualities. Unfortunately, our dataset did not allow us to consider different combinations of crop types and adjacent non-crop habitats or properties of the latter. More data, especially for the characterization of different adjacent habitats, are needed to shed light on such potential effects. Further, the effects of associated management practices (separately and in combination) on natural enemies need further investigation to gain a better mechanistic understanding of the drivers behind crop type associated distance functions revealed here. Our dataset focussed on the time frame in which the majority of the crop growth takes place between spring and crop harvest, while major benefits of adjacent habitats may occur at different time points, e.g. as refuges during field management and after harvest [31] and reservoirs for natural enemies earlier in the growing season [51]. The shape and strength of distance functions could change among these periods and further research is needed to understand spillover variations across time (see, e.g. [34]).

### (b) Potential causes of variation in distance functions

Our results, in part, contradict previously reported distance functions that varied between studies from increases over decreases to no changes for the same responses (see Introduction). What could cause such discrepancies? First, our results show that traits clearly shaped distance functions in carabids. Previous studies often did not differentiate between trophic groups or size classes (e.g. Anjum-Zubair *et al.* [24] or Pollier *et al.* [21]) and potential differences in trait compositions could have affected the observed distance functions. Second, distance functions could be affected by legacy effects of past crops in the crop rotation. Crops may vary in their suitability for carabid overwintering [36,53] and thus different initial assemblages emerging in the following crops can be expected. Third, apart from effects of adjacent woody or herbaceous habitats, different adjacent crop types could also affect distance functions as different crops often host distinct carabid assemblages [53]. Their potential effects deserve further attention. Fourth, even within the same crop type or species, management can vary considerably. Apart from obvious differences in management (conventional or organic) and tillage regimes (inversion or reduced tillage), other aspects such as pesticide applications or fertilization vary considerably depending on the local farm and field context. We are not aware of any studies addressing the effects of this heterogeneity in management practices on distance functions. Fifth, distance functions are likely to be affected by climatic conditions. Especially in dryer regions, vegetated and irrigated crop fields may attract arthropods, which may result in an accumulation in the crop fields and decay functions towards the field edges. Sixth, observed distance functions could be biased by sampling effort, i.e. the effects of spatial and/or temporal replication. A higher within-field replication increases the detection of rare species that occur at low densities or in narrow windows of activity. We also show that longer time frames of sampling yielded a better coverage of the total assemblage (electronic supplementary material, figure S3), probably due to phenological shifts within the assemblages. These increases in observed richness and related traits could influence distance functions if rare species are not equally distributed across space. Finally, our approach of pooling adjacent habitats into two types (herbaceous and woody) may have disguised potential beneficial effects of specific habitats. A better understanding of the interactions of various non-crop habitats (e.g. those established under agri-environmental schemes) with different adjacent crops is needed in order to determine their optimal characteristics and combinations to support natural pest and weed regulation in agricultural landscapes.

### (c) Implications for natural pest and weed regulation

The reported distance decay functions of carabids across most crop and adjacent habitat types has a strong potential to guide natural pest and weed regulation strategies. To minimize distance decays of natural pest regulation services, local conditions could be modified rather easily. Based on our synthesis, we recommend growing crop types with the strongest distance decays in the fields with the highest amount of edge area. As we found strong distance functions in most crop types and only limited effects of adjacent habitats, a general increase of field edge areas within agricultural landscapes could foster the magnitude and resilience of natural weed and pest regulation by avoiding distance decays and the resulting higher average carabid richness and densities (in granivorous species) at any point within the field, irrespective of the presence of adjacent non-crop habitats. This can be achieved in different ways: edge area could be increased by modifying the shape of the fields while maintaining their size, by a reduction of field sizes, e.g. by splitting large fields into smaller sections ideally also creating small buffer strips or linear landscape elements within fields that would simultaneously benefit overall biodiversity [7], or even by strip cropping [54], as effects were similar in control fields. Our findings support recent calls for a reduction in field sizes and an increase in edge density in agricultural landscapes [9,55]. Additionally, we see potential in crops that show no or shallow distance decays (e.g. legumes) to buffer distance decays in other crops (e.g. maize or vegetables) when grown (i) in sequence to foster within-field emergence in these crops in the next season or (ii) in mixed intercropping together (e.g. by legume intercropping). However, whether these strategies indeed compensate distance decays on natural enemies and their potential effects on crop yields needs to be investigated.

While it is difficult to change agricultural landscapes at the landscape scale with the many different stakeholders involved, changes at the field scale already hold large potential to increase natural pest and weed regulation by compensating for distance decays. Increased uptake and combinations of local measures such as the increase of edge area by the creation of buffer strips or linear landscape elements will generate more diverse and heterogeneous agricultural landscapes, and thus help to conserve overall biodiversity [55], and reduce the footprint of intensive agricultural production and reduce pesticide inputs in line with the farm-to-fork strategy of the European Green Deal.

## Data Availability

All data and code used for this manuscript are available via the Dryad Digital Repository: https://doi.org/10.5061/dryad.ns1rn8q0w [56]. Our analyses do not contain novel code. Electronic supplementary material is available online [57].
